# “Antigen Camouflage and Decoy” Strategy to Overcome Interference From Maternally Derived Antibody With Newcastle Disease Virus-Vectored Vaccines: More Than a Simple Combination

**DOI:** 10.3389/fmicb.2021.735250

**Published:** 2021-08-27

**Authors:** Zenglei Hu, Xiufan Liu

**Affiliations:** ^1^Joint International Research Laboratory of Agriculture and Agri-Product Safety, The Ministry of Education of China, Yangzhou University, Yangzhou, China; ^2^Animal Infectious Disease Laboratory, School of Veterinary Medicine, Yangzhou University, Yangzhou, China; ^3^Jiangsu Key Laboratory of Zoonosis, Yangzhou University, Yangzhou, China; ^4^Jiangsu Co-innovation Center for Prevention and Control of Important Animal Infectious Diseases and Zoonoses, Yangzhou University, Yangzhou, China

**Keywords:** antigen camouflage, antigen decoy, Newcastle disease virus, interference, maternally-derived antibody, vector vaccine

## Introduction

Vaccination is the most effective measure for the prevention and control of infectious diseases of humans and animals. Vaccines of different types have their advantages and disadvantages, and virus vectors represent a promising platform for the development of novel vaccines due to their outstanding strengths. Clinical trials of adenovirus 5-vectored vaccine against the newly-emerging severe acute respiratory syndrome coronavirus 2 (SARS-CoV-2) is a hallmark of virus-vectored vaccine (Zhu et al., 2020). In addition, in the field of veterinary medicine, some virus-vectored vaccines for poultry, such as the vaccines based on herpesvirus of turkey (HVT) (Palya et al., 2014) and fowlpox virus (Vagnozzi et al., 2012), are widely used worldwide and contribute to containment of major infectious diseases of poultry.

Newcastle disease virus (NDV), an important pathogen of poultry, is a single-stranded, negative-sense RNA virus, belonging to the genus *Avulavirus* in the family *Paramyxoviridae*. Several reviews have comprehensively summarized the research progress in NDV vector, confirming this virus as a promising vector for generation of novel vaccines for poultry, mammalian animals, and humans (Kim and Samal, 2016; Hu et al., 2020). In particular, some properties of NDV, such as high yield and good safety, allow mass administration *via* spray, drinking water and *in ovo* inoculation, endowing it a great potential to be used as a live vaccine vector for poultry industry. This advantage is of paramount importance to address the requirements of efficient disease control and early protection of some important infectious diseases for industrialized poultry production.

A lot of poultry vaccine candidates based on NDV vector were generated and they exhibited high efficacy in specific pathogen free (SPF) animals (Hu et al., 2020). However, in commercial chickens with the presence of maternally-derived antibody (MDA), the efficacy of NDV-vectored vaccines can be significantly impaired (Hu et al., 2020). Therefore, how to solve the problem of MDA interference is a primary challenge for developing effective NDV-vectored vaccines in the field. It is noted that MDA against both the vector and gene of interest (GOI) affects NDV-vectored vaccines. Some studies revealed that GOI-specific MDA even has a stronger inhibitory effect than vector-specific antibodies (Lardinois et al., 2016), which is actually largely overlooked by researchers. In the past decade, there has been significant progress in developing strategies to overcome or circumvent MDA interference with NDV-vectored vaccines. However, a comprehensive understanding of the mechanisms of MDA interference and novel strategies based on the related virology and vaccinology knowledge are still needed to generate efficacious NDV-vectored vaccines in the presence of MDA.

The aim of this opinion article was to present a novel strategy for generating effective NDV-vectored vaccines for poultry that may overcome MDA interference based on recent research advances in this field. This strategy combines engineering of both the vector (termed as “antigen camouflage”) and the GOI (termed as “antigen decoy”) to overcome MDA interference with NDV-vectored vaccines. Notably, integration of some factors regarding vaccination scheme, gene expression, and protein conformation were suggested to strengthen the effect of this combination.

## MDA Interference With NDV-Vectored Vaccines

MDA is a double-edged sword for poultry. On one hand, MDA affords early immunity to young offspring against major infectious diseases. On the other hand, the presence of MDA suppresses the effectiveness of vaccines administered in young poultry (Naqi et al., 1983; van Eck and Goren, 1991).

NDV vaccination is performed in almost all commercial poultry flocks worldwide. Undoubtedly, high level of NDV-specific MDA in young chicks can inhibit vaccine efficacy through multiple mechanisms, including virus neutralizing, Fc-mediated effector functions, B cell receptor (BCR) cross-linking, epitope masking, or B cell differentiation (Hu et al., 2020). Different lines of evidence have been obtained to support this phenomenon. In the presence of MDA against NDV, induction of the immune response by a prime or booster with a NDV-vectored H5 avian influenza virus (AIV) vaccine was suppressed at the time of vaccination (Bertran et al., 2018). Additionally, a NDV-vectored H5 vaccine conferred complete protection against H5N1 AIV challenge in SPF chickens, whereas it only provided 50% protection in NDV-MDA^+^ chickens (Steglich et al., 2013). Moreover, a higher dose of NDV-vectored H5N2 vaccine was required to overcome MDA against NDV and AIV to provide full protection against AIV and virulent NDV in commercial broilers (Sarfati-Mizrahi et al., 2010).

Besides MDA against the vector, pre-existing antibodies against the GOI inserted in NDV vector also exert a potent inhibitory effect on vaccine efficacy. To achieve high immunogenicity, the GOI is usually expressed in a membrane-anchored form to be incorporated efficiently into NDV particles. In this case, MDA against the GOI may affect the immune response elicited by the vectored vaccines. Lardinois et al. reported that H5-specific MDA had a stronger interference with a recombinant NDV-H5 vaccine compared to NDV-MDA (Lardinois et al., 2016). However, the mechanisms of GOI-specific MDA impairing vaccine efficacy remain largely obscure. First, GOI MDA may inhibit replication of the vectored vaccines *in vivo*. The GOI, especially the virus surface glycoproteins, incorporated into NDV virions may be also involved in virus attachment to the cells and virus life cycle. A recent study demonstrated that a recombinant NDV co-expressing soluble and membrane-bound H5 HAs replicated efficiently in chickens with high AIV-MDA titers, indicating no inhibition of AIV-MDA on vaccine virus replication (Murr et al., 2020a). However, there was no direct evidence elucidating the role of the GOI in replication of vectored vaccines. Second, binding of MDA with the GOI expressed in vaccine viruses or infected cells may result in formation of antigen-antibody immune complexes, activating Fc-dependent effector functions to clear the immune complexes. Third, BCR cross-linking, epitope masking and B cell differentiation involved in MDA interference with the vector may be also related to inhibition on the GOI. Thus, the functions of the GOI, an equally important arm of vectored vaccines, in replication of vaccine viruses and its interaction with MDA deserve more attention and efforts.

## “Antigen Camouflage” of the Vector to Avoid MDA Interference

**Figure 1:**
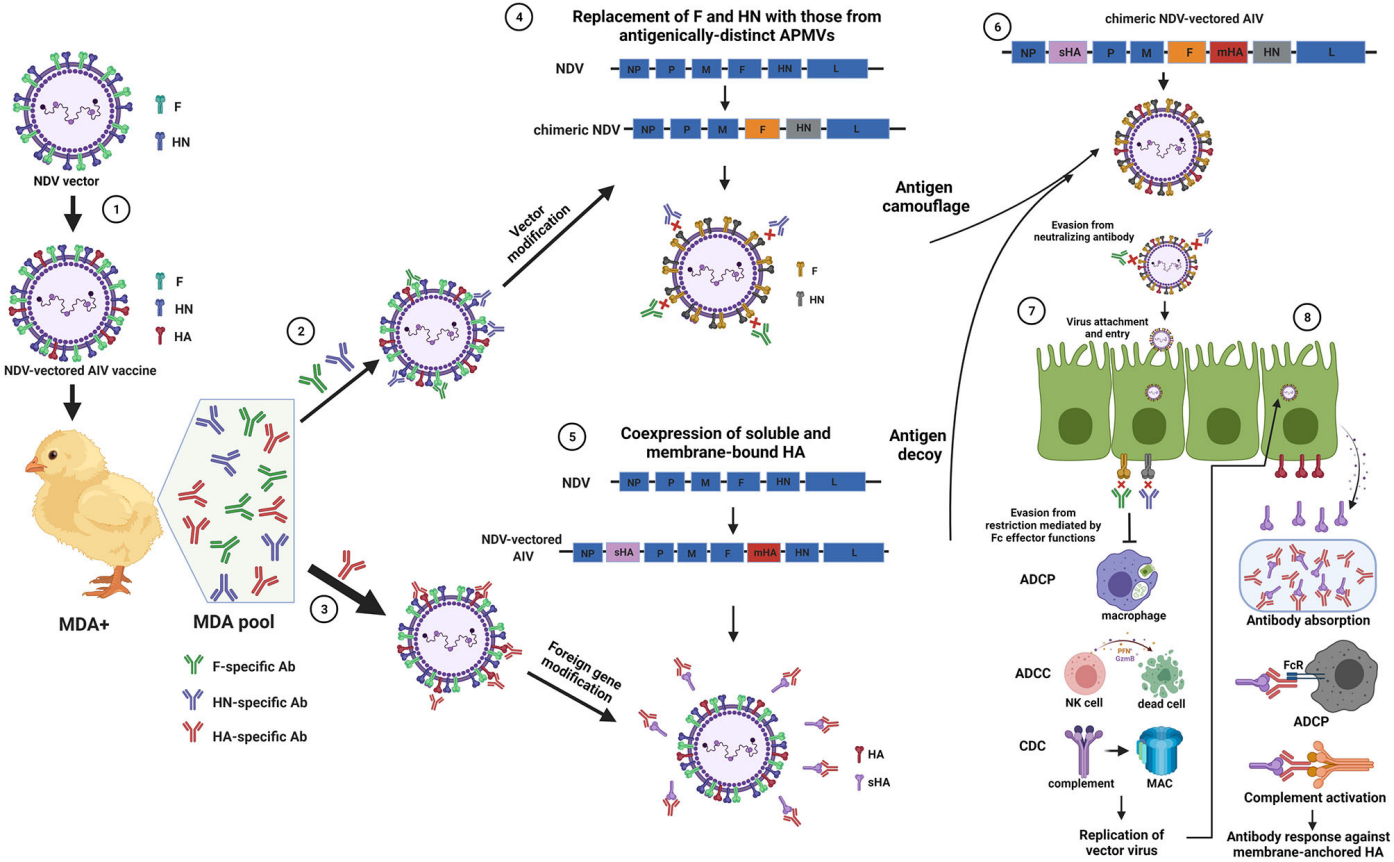
Schematic illustration of the “antigen camouflage” and “antigen decoy” combination in Newcastle disease virus vector to circumvent or overcome MDA interference. The hemagglutinin (HA) protein of influenza virus was shown as an example of the gene of interest. ① The traditional strategy of generating a recombinant NDV expressing the HA protein in a membrane-bound form. ② Maternally-derived antibody (MDA) against NDV binds the fusion (F) and hemagglutinin-neuraminidase (HN) proteins and interferes with antibody response. ③ HA-specific MDA binds the HA protein on virus surface and inhibits specific antibody immunity. A wider arrow (compared to ②) was used to indicate a stronger interference from the HA-MDA than NDV-MDA. ④ The “antigen camouflage” strategy with the F and HN genes in NDV replaced with those from antigenically-distant avian paramyxovirus viruses (APMVs). This modification results in evasion of the vectored vaccines from binding of NDV-MDA. Usage of entire APMVs as the vector can also achieve this purpose. ⑤ The “antigen decoy” strategy with co-expression of soluble and membrane-bound HA in NDV vector. This modification leads to production of soluble HA in the extracellular space, acting as a HA-MDA absorber and immunomodulator. sHA, soluble HA; mHA, membrane-bound HA. ⑥ Co-expression of both sHA and mHA in the chimeric NDV vector, combining the “antigen camouflage” and “antigen decoy” strategies together. ⑦ The resultant vaccine cannot be recognized by preexisting NDV antibodies, resulting in evasion from neutralization and antibody-dependent restriction effect, including antibody-dependent cellular phagocytosis (ADCP), antibody-dependent cell-mediated cytotoxicity (ADCC) and complement-dependent cytotoxicity (CDC). This allows efficient replication of vaccine in NDV-MDA chickens. NK cell, natural killer cells; PFN, perforin; GzmB, granzyme; MAC, membrane attack complex. ⑧ Replication of the vaccine in the cells (e.g. respiratory tract epithelial cells) produces both the mHA and sHA. The sHA may absorb a proportion of preexisting HA-specific antibodies and modulate Fc-mediated effector functions to stimulate antibody response against the mHA. The illustration was created with BioRender.com.

To solve the problem of MDA interference, modification of both the vector and GOI are equally important. Currently, an effective strategy of vector modification is to generate antigenically-distinct chimeric vectors or use avian paramyxoviruses (APMVs) of different serotypes as vectors, which is termed as the “antigen camouflage” strategy. AMPVs have similar genomic structure but diverse serotypes (AMPV-1 to−15 and APMV-17) (Jeong et al., 2018). NDV (APMV-1) has low cross-reactivity with other APMVs except for with APMV-3 (Nayak et al., 2012). Therefore, this feature was taken advantage to circumvent MDA interference with NDV vector. Chimeric vectors were generated by replacing the fusion (F) and hemagglutinin-neuraminidase (HN) genes in NDV with those from APMV-8 (Steglich et al., 2013) or APMV-2 (Kim et al., 2017; Liu et al., 2018). H5N1 and H9N2 subtype AIV vaccine candidates based on these chimeric vectors induced strong immune response and provided good protection against AIV infection in chickens with NDV MDA. In addition, APMVs of other serotypes, including APMV-2,−6, and−10, can also serve as potential vaccine vectors in commercial chickens with antibodies against NDV (Tsunekuni et al., 2017). Of note, the vaccines based on such vectors have a great potential to be used for emergency vaccination during outbreaks of infectious diseases, such as highly pathogenic avian influenza. Mechanistically, the “antigen camouflage” strategy leads to evasion of antibody-mediated restriction, allowing efficient replication of the vectors in MDA-positive chickens (Figure 1). On one hand, neutralizing antibodies, mainly against the F and HN proteins, significantly inhibit virus replication by blocking attachment of NDV to cellular receptors. Chimeric vectors carrying distinct F and HN genes can escape such neutralizing effect exerted by NDV antibodies and successfully attach the host cells for replication. On the other hand, non-neutralizing antibodies also restrict virus replication through clearance of virus-infected cells or virus particles *via* Fc-dependent effector functions, including antibody-dependent cell-mediated cytotoxicity (ADCC), antibody-dependent cellular phagocytosis (ADCP), and complement-dependent cytotoxicity (CDC). Replacement of the F and HN genes in chimeric vectors may abrogate antigen-antibody binding and thus facilitate vector viruses to evade such restriction of replication.

## The “Antigen Decoy” Strategy to Overcome MDA Interference With the GOI

### Many Viruses Employ the “Antigen Decoy” Strategy for Immune Evasion

The “antigen decoy” strategy is an effective immune evasion tactic used by many viruses (Cook and Lee, 2013). These viruses express their main antigenic glycoproteins in two forms, the full-length (FL), membrane-anchored and truncated (secreted or soluble) version. The truncated proteins are secreted into the extracellular space, acting as the “antigen sink” or “antigen decoy” to absorb specific antibodies and modulate the host immune response, assisting the virus to escape the restriction from the host. For example, in addition to the membrane-bound glycoprotein (GP), Ebola virus (EBOV) also produces a large amount of secreted GP (sGP) through ectodomain shedding (Dolnik et al., 2004). EBOV sGP absorbs pre-existing GP antibodies and subverts the host antibody immune response to induce cross-reactivity with the epitopes it shares with the membrane-bound GP, which promote virus immune evasion (Mohan et al., 2012). Similarly, shedding of soluble GP1 of Lassa virus can be detected in serum of patients during acute infection, suggesting that this soluble isoform of GP1 may mediate immune evasion of the virus (Branco et al., 2010). The secreted GP of respiratory syncytial virus (RSV) acts as an antigen decoy for host antibodies and can regulate immunity *via* interaction with Fc receptor bearing leukocytes (macrophages) and complements (Bukreyev et al., 2008, 2012). The critical role of secreted antigens in promoting viral immune evasion underlies the employment of the “antigen decoy” strategy in NDV-vectored vaccines.

### Expression of Soluble Antigens in NDV Vector as Decoys

Compared to vector optimization, modifications of the GOI in NDV vector to avoid MDA interference are limited. It is generally accepted that efficient incorporation of the GOI into NDV particles is a prerequisite to achieve high immunogenicity, which may partly stop researchers from engineering the GOI. However, recent results verified that modification of GOI expression pattern is an effective means to overcome the inhibition by GOI MDA. Soluble and membrane-bound H5 HAs were co-expressed in a recombinant NDV (rNDVsolH5_H5), resulting in a significant increase in antigen levels (Murr et al., 2020b). They speculated that elevated antigen levels may enhance antigenicity of HA, which may overwhelm the interference of AIV-MDA. In a following study, AIV-MDA^+^ chickens at different ages were vaccinated with the rNDVsolH5_H5 and the vaccine provided full protection against AIV challenge in 3-week-old chickens (Murr et al., 2020a). The vaccine provided 40 and 85% protection in younger chickens with higher AIV-MDA at 1 and 2 weeks post hatch, while it still strongly reduced shedding of challenge virus. Another critical finding from their study is that the vaccine virus replicated efficiently in AIV-MDA^+^ chickens, leading to an assumption that poor protection in younger chickens are caused by an H5 antigen-specific block, rather than by MDA interference with the vaccine virus. According to the role of secreted glycoproteins in virus survival, we speculated that the soluble GOI expressed by NDV vector may antagonize MDA interference through two mechanisms (Figure 1). First, soluble antigens, acting as an “antigen decoy”, may absorb a proportion of MDA, which decrease the amount of active antibodies binding to membrane-bound antigens. Second, as reported in previous RSV studies, soluble antigens may interfere with the influx or functioning of complement and Fc receptor bearing immune cells, such as macrophages, natural killer cells and neutrophils, that contribute to virus clearance by ADCC, ADCP, and CDC. This may spare membrane-bound antigens from antibody-dependent restriction, promoting specific immunity against the GOI. However, little is known about the role of soluble proteins (or antigens) in virus life cycle (or immunogenicity of viral-vectored vaccines) and the related mechanisms, which deserve more attention and efforts.

### Efficacy of Virus Vectors Expressing Soluble Antigens: No Consensus Yet

Unlike the defined role of secreted antigens in virus survival, it is controversial about their immunogenicity when expressed in virus vectors as immunogens. On one hand, different virus vectors expressing soluble protective antigens exhibited high immunogenicity and efficacy in animals, comparable to their counterparts expressing membrane-anchored antigens. The soluble F protein of RSV or the HA head (secreted) expressed in adenovirus backbone provided solid protection against virus infection (Kim et al., 2013; Fu et al., 2014). The glycoprotein D of bovine herpesvirus 1 or glycoprotein G of nipah virus expressed in modified Ankara virus (MVA) vector in the secretion form induced protective immunity in animal models (Zajac et al., 2017; Kalodimou et al., 2020). Recombinant measles viruses (MV) expressing the FL spike (S) protein of the Middle East respiratory syndrome coronavirus (MERS-CoV) or a truncated variant elicited robust neutralizing antibodies and T cell immunity in mice (Malczyk et al., 2015). On the other hand, the MVA expressing membrane-anchored pre-fusion S but not secreted S1 induced strong neutralizing antibody response against SARS-CoV-2 in mice (Routhu et al., 2021), which is quite different from the findings observed for MV-vectored MERS-CoV. Our previous study revealed that the HA ectodomain of H7N9 subtype AIV expressed in a NDV vector failed to elicit detectable antibodies in chickens (Hu et al., 2018b). Furthermore, Oreshkova et al. expressed both the entire HA and soluble ectodomain in Rift Valley fever virus and only the entire form conferred protection against a lethal challenge (Oreshkova et al., 2014). These conflicting observations may be associated with the properties of the vector, the immunization route or animal models.

Notably, some interesting findings were gained for recombinant NDVs expressing soluble HA. Cornelissen et al. generated two recombinant NDVs expressing the FL (NDV-H5) and a soluble trimeric HA (NDV-sH5) of H5N1 subtype AIV (Cornelissen et al., 2012). A single intramuscular immunization with these two vaccines fully protected chickens against H5N1 challenge and reduced virus shedding. However, NDV-sH5 only provided 50% protection rate when administered *via* respiratory tract, which was lower than that for NDV-H5 (80%). Similarly, the FL HA and HA1 (secreted) of H5N1 AIV were expressed in a thermostable NDV and low protection was observed for both vaccines in chickens and mice, despite they elicited high levels of H5N1-specific antibody response (Xu et al., 2020). However, addition of the tissue plasminogen activator signal sequence to the HA1 marked enhanced protection. The recombinant NDV expressing the secreted trimeric S ectodomain of infectious bronchitis virus can protect against clinical signs and tracheal lesions but not virus shedding, even at a high dose of 10^7^ 50% embryo infectious dose (Zegpi et al., 2020). Co-expression of chicken granulocyte-macrophage colony-stimulating factor in this recombinant virus significantly reduced tracheal viral load and tracheal lesions (Khalid et al., 2021). An earlier report also showed that addition of a dendritic cell-targeting single-chain Fv to the Gag protein of HIV significantly strengthened antibody and T cell immunity as well as protection against virus challenge (Maamary et al., 2011). Collectively, the results of NDV vector expressing antigens in different forms indicate that antigens expressed in the FL, membrane-bound form is critical to achieve high protection of the vaccines.

## Considerations of Strengthening the Effect of “Antigen Camouflage and Decoy” Combination

Since the “antigen camouflage” and “antigen decoy” strategies give promising results in chickens with MDA against the vector or GOI, respectively, it is reasonable to combine these two arms in NDV vector to avoid MDA interference. However, no such studies were performed to evaluate the effect of this combination on the immunogenicity and efficacy of the vectored vaccines in the presence of MDA against both the vector and GOI. Additionally, Murr et al. revealed that the NDV vector co-expressing soluble and membrane-bound H5 HA only provided 40% protection in 1-week-old chickens with high HI antibody titers of ~4 log_2_ (Murr et al., 2020a), indicating that further elevation of HA yield may be required to overcome high levels of MDA. Therefore, some points should be considered to optimize the effect of this combination.

First, vaccination programs and serological surveillance of the target flocks should be optimized to enhance the effectiveness of “antigen camouflage” vaccines. There are two attractive merits of this strategy: confer early protection to young chickens *via* mass administration against certain diseases, such as avian influenza, in the presence of MDA; allow emergency vaccination in the case of disease outbreaks. However, one limitation of “antigen camouflage” vaccines is the deficiency in providing early protection against NDV. Thus, vaccination programs should be optimized by including other vaccines to compensate NDV immunity. For example, the commercial HVT-vectored vaccines, i.e., Vectormune® ND, that is not affected by MDA can be administered to provide early protection against NDV and “antigen camouflage” vaccines can be administered at 1- or 2-week-old to confer protection against the target disease. Another limitation is that “antigen camouflage” vaccines are still sensitive to MDA against the substitutive antigens, although they can evade NDV-specific immunity. For example, if NDV-APMV8 chimeric virus is used as the vaccine vector, pre-existing antibodies against APMV8 may still interfere with the vaccine efficacy because these antibodies can be detected in commercial chicken flocks (Warke et al., 2008). Therefore, it is essential to determine the prevalence of antibodies against main APMVs in the target flocks and tailor “antigen camouflage” vaccines by expressing antigens distinctive from the prevalent APMVs.

Second, expression levels of the soluble and membrane-anchored antigens and vaccine virus replication should be balanced. Sufficient amounts of the antigens in these two forms are required to absorb high proportions of MDA and to stimulate protective antibody immune response, respectively. In Murr's work, the soluble and FL HA were inserted between the P-M and F-HN genes, which resulted in 5.25-fold increase in total HA expression level (Murr et al., 2020b). Other combinations of the insertion sites in NDV genome for the GOI should be further evaluated. Additionally, Tsunekuni et al. found that flanking of the H5 HA gene with the 5′ and 3′-untranslated regions of APMV-10 remarkably elevated HA protein level expressed by APMV-10 vector and the resultant vaccine candidates provided complete protection and significantly inhibited virus shedding (Tsunekuni et al., 2020). This strategy can be also used to increase expression levels of both the soluble and membrane-bound GOI in chimeric or APMV vectors. Moreover, due to low capacity of NDV genome, the GOI can be expressed using the internal ribosome entry site strategy to maintain efficient replication of the vaccine virus *in vivo* (Zhang et al., 2015; Hu et al., 2018a).

Third, modification of the soluble GOI should be considered to enhance their “antigen decoy” activity. Natural conformations, such as trimer, are vital for biofunctions of many viral surface glycoproteins. Therefore, protein trimerization signals are needed to restore the natural conformation of the GOI when the transmembrane domain is deleted to make the soluble protein. The truncated HA of H5N1 AIV was expressed in trimer in NDV vectors through adding an isoleucine-zipper trimerization motif GCN4 to the C-terminal (Cornelissen et al., 2012; Murr et al., 2020b). In addition, NDV-vectored vaccines have tissue tropism mainly in the respiratory tract and the soluble proteins secreted into the extracellular space cross the mucosal barrier to enter systematic circulation inefficiently due partly to the physical properties of the epithelial cells. Fusion of the antigen with the Fc domain of IgG is a feasible means to enhance transcytosis of the antigen (Ye et al., 2011). Interaction between the Fc-antigen and the neonatal FcR (FcRn) expressed in the epithelial mucosa facilitates the delivery of the antigen across the mucosal barrier and increase half-life of the antigen in serum (Gosselin et al., 2009; Ye et al., 2011), which may strengthen the role of the soluble GOI in neutralizing MDA.

## Summary

MDA interference with NDV-vectored vaccines is sophisticated, with involvement of antibodies against both the vector and GOI. Based on a summary of the related knowledge in virology and vaccinology, we propose a novel strategy combining the “antigen camouflage” and “antigen decoy” to overcome MDA interference with the vector and GOI. Of note, these two strategies are not combined simply and several factors regarding vaccination program, serological background, protein expression level, virus replication, and activity of the “soluble decoy” should be considered to optimize this strategy. Therefore, the present article added new information for the development of effective NDV-vectored vaccines.

## Author Contributions

ZH wrote the manuscript. XL critically revised the manuscript. All authors approved the submission of the manuscript.

## Conflict of Interest

The authors declare that the research was conducted in the absence of any commercial or financial relationships that could be construed as a potential conflict of interest.

## Publisher's Note

All claims expressed in this article are solely those of the authors and do not necessarily represent those of their affiliated organizations, or those of the publisher, the editors and the reviewers. Any product that may be evaluated in this article, or claim that may be made by its manufacturer, is not guaranteed or endorsed by the publisher.
